# A Delphi consensus statement for digital surgery

**DOI:** 10.1038/s41746-022-00641-6

**Published:** 2022-07-19

**Authors:** Kyle Lam, Michael D. Abràmoff, José M. Balibrea, Steven M. Bishop, Richard R. Brady, Rachael A. Callcut, Manish Chand, Justin W. Collins, Markus K. Diener, Matthias Eisenmann, Kelly Fermont, Manoel Galvao Neto, Gregory D. Hager, Robert J. Hinchliffe, Alan Horgan, Pierre Jannin, Alexander Langerman, Kartik Logishetty, Amit Mahadik, Lena Maier-Hein, Esteban Martín Antona, Pietro Mascagni, Ryan K. Mathew, Beat P. Müller-Stich, Thomas Neumuth, Felix Nickel, Adrian Park, Gianluca Pellino, Frank Rudzicz, Sam Shah, Mark Slack, Myles J. Smith, Naeem Soomro, Stefanie Speidel, Danail Stoyanov, Henry S. Tilney, Martin Wagner, Ara Darzi, James M. Kinross, Sanjay Purkayastha

**Affiliations:** 1 Department of Surgery and Cancer, Imperial College, London, UK; 2 Institute of Global Health Innovation, Imperial College London, London, UK; 3 Department of Ophthalmology and Visual Sciences, University of Iowa, Iowa City, IA USA; 4 Department of Electrical and Computer Engineering, University of Iowa, Iowa City, IA USA; 5 Department of Gastrointestinal Surgery, Hospital Clínic de Barcelona, Barcelona, Spain; 6 Universitat de Barcelona, Barcelona, Spain; 7 CMR Surgical Limited, Cambridge, UK; 8 Newcastle Centre for Bowel Disease Research Hub, Newcastle University, Newcastle, UK; 9 Department of Colorectal Surgery, Newcastle Hospitals, Newcastle, UK; 10 Department of Surgery, University of California, Davis, CA USA; 11 Department of Surgery and Interventional Sciences, University College London, London, UK; 12 Department of General and Visceral Surgery, University of Freiburg, Freiburg im Breisgau, Germany; 13 Faculty of Medicine, University of Freiburg, Freiburg im Breisgau, Germany; 14 Division of Computer Assisted Medical Interventions (CAMI), German Cancer Research Center (DKFZ), Heidelberg, Germany; 15 Solicitor of the Senior Courts of England and Wales, Independent Researcher, Bristol, UK; 16 Endovitta Institute, Sao Paulo, Brazil; 17 FMABC Medical School, Santo Andre, Brazil; 18 The Malone Center for Engineering in Healthcare, The Johns Hopkins University, Baltimore, MD USA; 19 Department of Computer Science, The Johns Hopkins University, Baltimore, MD USA; 20 Department of Vascular Surgery, University of Bristol, Bristol, UK; 21 LTSI, Inserm UMR 1099, University of Rennes 1, Rennes, France; 22 Otolaryngology, Head & Neck Surgery and Radiology & Radiological Sciences, Vanderbilt University Medical Center, Nashville, TN USA; 23 International Centre for Surgical Safety, Li Ka Shing Knowledge Institute, St. Michael’s Hospital, University of Toronto, Toronto, ON Canada; 24 Stryker Corporation, Kalamazoo, MI USA; 25 Faculty of Mathematics and Computer Science, Heidelberg University, Heidelberg, Germany; 26 Medical Faculty, Heidelberg University, Heidelberg, Germany; 27 LKSK Institute of St. Michael’s Hospital, Toronto, ON Canada; 28 Department of Surgery, Hospital Clínico San Carlos, Madrid, Spain; 29 Fondazione Policlinico Universitario A. Gemelli IRCCS, Rome, Italy; 30 IHU-Strasbourg, Institute of Image-Guided Surgery, Strasbourg, France; 31 ICube, University of Strasbourg, Strasbourg, France; 32 School of Medicine, University of Leeds, Leeds, UK; 33 Department of Neurosurgery, Leeds Teaching Hospitals NHS Trust, Leeds, UK; 34 Department of General, Visceral and Transplantation Surgery, Heidelberg University Hospital, Heidelberg, Germany; 35 National Center for Tumor Diseases, Heidelberg, Germany; 36 Innovation Center Computer Assisted Surgery (ICCAS), Universität Leipzig, Leipzig, Germany; 37 Department of Surgery, Anne Arundel Medical Center, School of Medicine, Johns Hopkins University, Annapolis, MD USA; 38 Department of Advanced Medical and Surgical Sciences, Università degli Studi della Campania “Luigi Vanvitelli”, Naples, Italy; 39 Colorectal Surgery, Vall d’Hebron University Hospital, Barcelona, Spain; 40 Department of Computer Science, University of Toronto, Toronto, ON Canada; 41 Vector Institute for Artificial Intelligence, Toronto, ON Canada; 42 Unity Health Toronto, Toronto, ON Canada; 43 Surgical Safety Technologies Inc, Toronto, ON Canada; 44 Faculty of Future Health, College of Medicine and Dentistry, Ulster University, Birmingham, UK; 45 Department of Urogynaecology, Addenbrooke’s Hospital, Cambridge, UK; 46 University of Cambridge, Cambridge, UK; 47 The Royal Marsden Hospital, London, UK; 48 Institute of Cancer Research, London, UK; 49 Department of Urology, Newcastle Upon Tyne Hospitals NHS Foundation Trust, Newcastle upon Tyne, UK; 50 Division of Translational Surgical Oncology, National Center for Tumor Diseases (NCT/UCC), Dresden, Germany; 51 Centre for Tactile Internet with Human-in-the-Loop (CeTI), TU Dresden, Dresden, Germany; 52 Wellcome/ESPRC Centre for Interventional and Surgical Sciences, University College London, London, UK; 53 Department of Colorectal Surgery, Frimley Health NHS Foundation Trust, Frimley, UK

**Keywords:** Medical ethics, Health policy, Surgery

## Abstract

The use of digital technology is increasing rapidly across surgical specialities, yet there is no consensus for the term ‘digital surgery’. This is critical as digital health technologies present technical, governance, and legal challenges which are unique to the surgeon and surgical patient. We aim to define the term digital surgery and the ethical issues surrounding its clinical application, and to identify barriers and research goals for future practice. 38 international experts, across the fields of surgery, AI, industry, law, ethics and policy, participated in a four-round Delphi exercise. Issues were generated by an expert panel and public panel through a scoping questionnaire around key themes identified from the literature and voted upon in two subsequent questionnaire rounds. Consensus was defined if >70% of the panel deemed the statement important and <30% unimportant. A final online meeting was held to discuss consensus statements. The definition of digital surgery as the use of technology for the enhancement of preoperative planning, surgical performance, therapeutic support, or training, to improve outcomes and reduce harm achieved 100% consensus agreement. We highlight key ethical issues concerning data, privacy, confidentiality and public trust, consent, law, litigation and liability, and commercial partnerships within digital surgery and identify barriers and research goals for future practice. Developers and users of digital surgery must not only have an awareness of the ethical issues surrounding digital applications in healthcare, but also the ethical considerations unique to digital surgery. Future research into these issues must involve all digital surgery stakeholders including patients.

## Introduction

Digital technologies ranging from robotics^1^, virtual and augmented reality^2^, and artificial intelligence (AI)^3^ offer the promise of data-driven precision surgery with the ultimate goal of improving patient outcomes, operative performance and surgeons and their teams’ productivity and efficiency^4,5^. The uptake of digital technology is not limited to the operating room alone; digital technology now has a role in areas as diverse as preoperative planning^6^, surgical risk prediction^7^, and surgical performance assessment^8,9^.

Much of the rapid adoption of these technologies is being driven by commercial opportunity and the promise of better outcomes for surgeons and patients. The surgical robotics market, which makes up only a proportion of the global digital surgical technologies market, has been valued at $5 billion with an estimated value of $16.77 billion by 2031^10^. Surgeons and healthcare providers therefore have increasing freedom to choose which technologies to incorporate into their operating rooms.

Despite this emerging role of digital technology within surgery, the definition of the term digital surgery, however, remains unclear. Defining digital surgery is of significant importance for a number of reasons. Firstly, surgery is a high-risk clinical intervention, and failures in these technologies have the potential to cause serious harm. Surgery is unlike other medical use cases as it is dependent on real-time analysis of heterogenous data, and patients and surgeons deserve standardisation of emerging technologies for risk mitigation. Secondly, digital surgery cannot be effectively trialled or understood unless there is clarity of definition. It is impossible to quality assure clinical interventions or trials without this. As digital surgery is rapidly incorporated into clinical practice, it is also essential that we are able to explain digital surgery to patients clearly and consistently, especially in the context of data collection and processing for digital surgery applications. Finally, the lack of clarity in digital surgery impedes progress. Rapidly emerging fields such as digital surgery require clarification of research priorities and areas for collaboration.

Surgery is not unique amongst medical specialties in its increasing use of digital technology. However, digital surgery will incorporate not only the use of digital technology within surgery but also the revolution in the culture and practice of surgery, a specialty which has historically focused on post-operative outcomes with little emphasis on data collection within the operating room. As a result, the potential benefits and risks of the incorporation of digital technologies are unique to surgery, necessitating digital surgery to be set apart from other specialties and clearly defined.

The use of digital technology in surgery may pose risks which are not communicated to patients as part of current consent practices. Beyond the established risks of introducing novel technologies into clinical environments, digital technologies are often dependent on the large-scale processing of personalised data which poses specific ethical and data governance challenges. A state of the nation survey into AI in healthcare in 2018 showed that 88% of respondents viewed the building of an ethical framework to build/preserve trust and transparency and 82% of respondents viewed clarity about ownership of data as a very or extremely important enabler for AI^11^. Digital applications must therefore not only be accurate to succeed, but be based on an ethical framework^12^.

Lessons can be learned from AI ethics across other sectors. Global AI ethical guidelines converge to 5 core themes: transparency; justice and fairness; non-maleficence; responsibility; and autonomy^13,14^. These themes are able to provide broad guidance for those developing and utilising digital tools in surgery but there is a lack of guidance to cover specific ethical and data governance issues related to the practice of surgery. The UK’s NHS AI lab has published governance and data frameworks for the safe adoption of AI systems in healthcare^15^ and emerging guidance concerning ethics and AI in healthcare has also recently been published by the World Health Organisation^16^. This provides an overall framework for AI in healthcare but does not address specific issues of AI ethics in surgery. Firstly, surgical decision making is unique, requiring quick decisions which are highly contextual and on which the patient often cannot be consulted. Secondly, datasets across surgery are extensive and heterogenous including surgical videos, sensor data and teamwork data^17,18^. Data governance issues across surgery affect not only the patient but also the surgeon and the wider surgical team. The recent publication of an ethical framework for the use of AI in robotic surgical training^19^ signals the specific nature of surgical practice and the need for ethical issues within digital surgery to be explored. In addition, surgical data ownership is controversial, and does not fit in existing legal frameworks.

There has been little work in the published literature concerning the ethical and data governance issues concerning digital technology in surgery. In areas which span multiple areas of expertise and where there is insufficient information, consensus methods such as the Delphi technique have been effectively employed^20^. Therefore, we conducted a Delphi exercise to firstly generate key ethical and data governance issues across digital surgery and secondly to correlate these views across key stakeholders within digital surgery to reach consensus. The aims of the study are firstly to agree a consensus definition for the term digital surgery which can be utilised both in clinical and academic settings, secondly to determine important ethical and data governance issues surrounding digital surgery, and finally to identify key barriers and research goals for the future of digital surgery.

## Results

52 expert panellists completed Round 1, 44 panellists (84.6%) completed Round 2 and 38 (86.4%) panellists completed Round 3. 20 members of the public also participated in Round 1 and issues generated in this round were combined with expert-generated issues into Round 2. Cronbach’s alpha, was 0.981 and 0.881 in Rounds 2 and 3 respectively indicating high inter-rater reliability. The full questionnaire and results of Rounds 2 and 3 can be found in Supplementary Notes 2 and 3. Consensus was obtained across 114 issues which were grouped into 7 key areas: definition of digital surgery; data; privacy, confidentiality and public trust; consent; law; litigation and liability; and commercial partnerships. Consensus was reached on 38 barriers associated with the development, deployment and monitoring of digital surgical systems and 22 technical, clinical and organisational future research goals for digital surgery. A list of all consensus issues can be found in Supplementary Note 4.

### Defining digital surgery

**Fig. 1:**
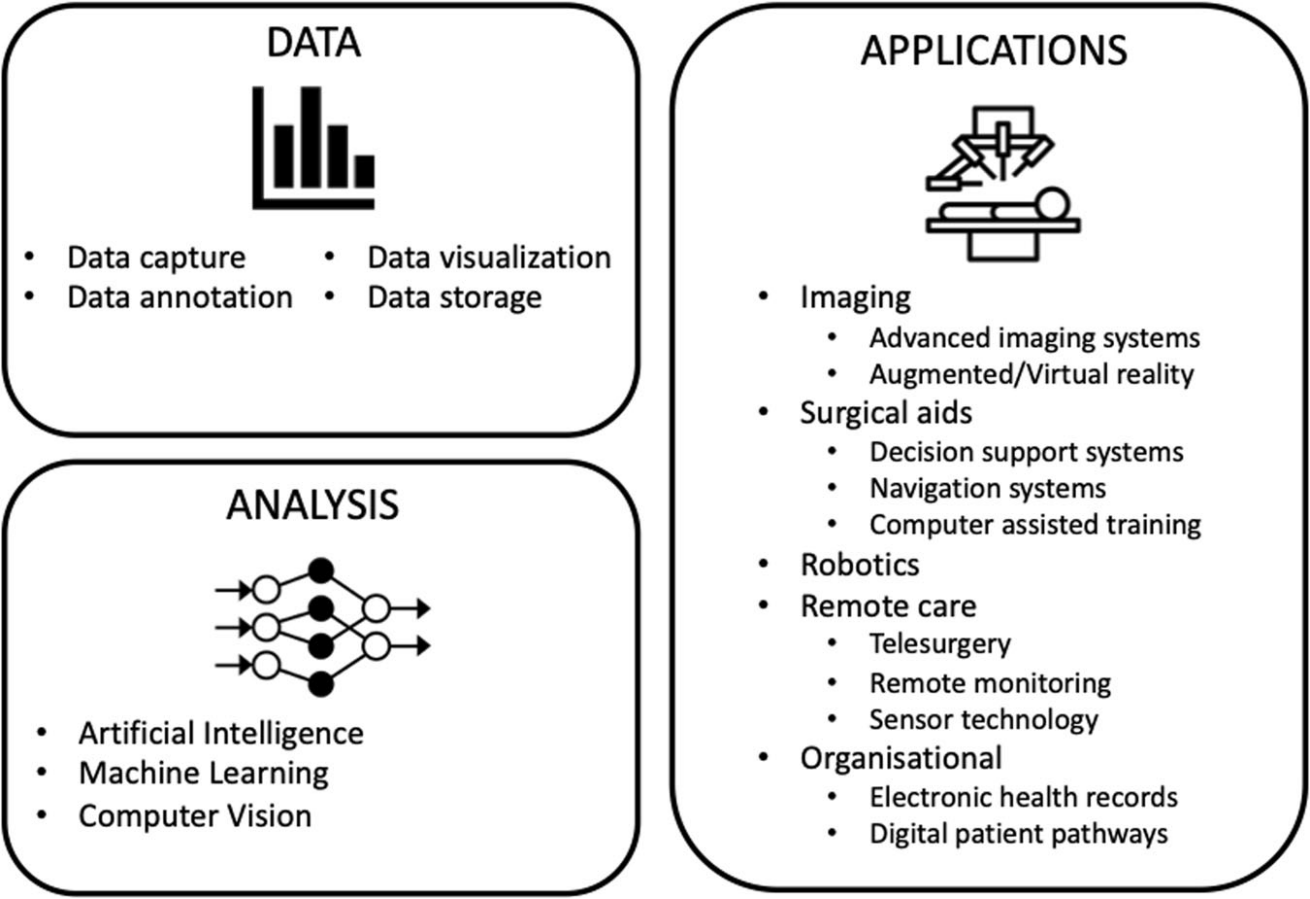
Elements of digital surgery identified by the Delphi panel. Consensus elements were grouped into three themes: data; analysis; and applications.

71% of participants agreed that, at present, the definition of digital surgery is unclear. 86% of participants agreed that digital surgery should incorporate pre-operative, peri-operative and post-operative aspects of surgery. 82% of participants agreed that the term digital surgery should include not only operative aspects of surgery but also to other aspects including training, diagnosis and investigation. Participants were invited to propose and comment on definitions of the term digital surgery. This definition was discussed in the final online meeting with 100% of panellists in agreement of the final consensus definition (TEXTBOX 1). This definition provides a practical definition which can be adopted both in clinical and research purposes and by those with limited knowledge of the field. Panellists were also encouraged to agree on the technologies that comprise digital surgery (Fig. 1). Finally, panellists agreed to the existing and potential benefits of digital surgery (Table 1).

**Table 1:** Benefits of digital surgery identified by the Delphi panel.

Patient	Surgeon	Organisation
Improving clinical outcomes Improving patient care Improving diagnostics Delivering patient specific treatment Identifying patient deterioration more promptly	Allowing pre-operative treatment planning Providing decision support to the surgeon Reducing cognitive load on the surgeon Automating surgical processes Error prediction Error detection Standardising surgical processes Improving surgeon ergonomics and health Evaluating surgeon performance Accelerating surgical education	Improving surgical efficiency Improving cost efficiency Quantifying outcome beyond survival and other standard outcome measures Understanding benefits and limitations of surgical strategies Understanding and improving team dynamics

TEXTBOX 1 Definition of digital surgery “Digital surgery is defined as the use of technology for the enhancement of preoperative planning, surgical performance, therapeutic support, or training, to improve outcomes and reduce harm.”

### Data issues

#### Data access

Data is central both to the development and use of digital surgical technologies. Panellists agreed that there is a current lack of infrastructure for data acquisition, and that a significant contributor to this is the lack of interoperability (previously defined as the ability of two or more systems or components to exchange information and to use the information that has been exchanged^21^) between different devices and systems. Panellists agreed, therefore, that data is not readily available in a digital format. Furthermore, they concluded that there is a lack of reliable datasets available and this is made more difficult due to the lack of data quality, data annotation, and data formatting standards. Finally, panellists agreed that determining appropriate access to data was an important issue and that governance processes at present were overcomplicated and obstructive.

#### Data storage and security

For digital surgery to succeed, data must be appropriately and securely stored. Panellists agreed that hospitals currently lack the technical ability and structure for appropriate data storage. Panellists also highlighted that the costs of data storage and appropriate encryption of data are important issues. Moreover, panellists agreed that institutions are not equipped and under resourced to perform appropriate cybersecurity. Lastly, the implications of data breaches are currently poorly defined.

#### Data sharing

Data sharing encompasses sharing of data including between different technologies, between hospitals, and between hospitals and commercial partners. Panellists agreed that there are currently no guidelines concerning data ownership, and the international legal requirements concerning data sharing are unclear. Furthermore, panellists agreed that adherence to present data rules can hamper competitiveness. Moreover, data sharing across international boundaries is problematic and there is no consensus on data sharing formats. Finally, panellists agreed that there is a lack of motive for surgeons to share data.

### Privacy, confidentiality and public trust

Preservation of privacy and confidentiality is essential, not only to safeguard patient autonomy but also to ensure patient trust which is vital for future development of digital surgical applications. Panellists agreed that appropriate anonymisation of data was of significant importance. Moreover, consensus was reached that patient agreements for data sharing should be determined and that data should be used explicitly for the purposes it was collected for. Panellists agreed that there is a lack of education among surgical teams about the significance of data they are collecting; this suggests that there is a need for greater education around data governance and security.

High profile misuse of personal data both in a clinical and non-clinical setting has led to concern amongst the public concerning personal data collection, especially when in collaboration with a commercial entity^22^. Panellists agreed that ensuring public trust on data sharing was an important issue, that there has been a lack of engagement with the public to date, and that there is a general lack of education around AI among the public. Panellists agreed the following important issues affect public trust: the lack of explainability due to the opaqueness of surgical AI systems; the fear of AI reinforcing biases in datasets; and the failure to produce an effective surgical AI system to date. Regardless of success or failure, panellists agreed that there should be mandatory reporting of outcomes. Privacy and confidentiality issues extend beyond the patient to the wider surgical team; panellists agreed that the surgeon’s right to privacy and the potential influence of digital surgical systems on their behaviour was an important issue.

### Consent

Surgical data pipelines are vital for development and evaluation of digital surgical technologies. Appropriate consent must be sought from patients to ensure patient autonomy and privacy. According to the UK and EU’s General Data Protection Regulation (GDPR), consent must be specific and informed and therefore must include the purposes of the processing and the right to withdrawal at any time. When applied to a digital surgical context, panellists agreed that issues may arise with consent procedures when collecting data for unknown future applications. Furthermore, panellists agreed that an important issue was the management of a patient who chooses to withdraw consent. The ‘right to erasure’, albeit qualified, is problematic in the context of digital surgery; for example, for AI systems previously trained on the dataset of a patient who chooses to withdraw their consent.

It is unclear to what extent patients should be informed when consenting to share their data. Panellists agreed that it is important that patients fully understand what is being asked when consenting for data sharing, and that educating patients about data sharing for digital surgical applications should be a priority. Panellists agreed that issues of differing requirements of consent between countries was of importance. Finally, panellists agreed that the rights of the surgeon and the wider surgical team to opt out of data collection should be considered.

Panellists, therefore, came to consensus that there should be a standardised methodology for consenting patients to share their data for digital surgery applications. Panellists also agreed that digital surgery consent procedures should (1) delineate the extent of data collection, (2), delineate who will access the data, (3) explain why the data will be collected, (4) allow data collection for future or unknown applications, and (5) patients should consent separately should commercial partners have access to their data.

### Law

The key legislation within Europe governing the use of data within digital surgery is the UK and EU GDPR. This overarching piece of legislation governs health data (as well as other data) irrespective of format or how it is collected. GDPR is technology neutral with no mention of AI or associated technologies. However, significant focus is given to large-scale processing of personal data.

US law is more complex. The majority of the relevant legislation is governed within the Privacy Rule within the Health Insurance Portability and Accountability Act (HIPAA). In contrast to GDPR, HIPAA is more restricted and concerns itself only with protected health information (PHI) which is identifiable. Data which has been de-identified is therefore not governed by HIPAA. Furthermore, data ownership under HIPAA is an issue which is yet to be resolved.

Panellists agreed that an important issue was the lack of standardisation of terminology concerning AI in law and the lack of dedicated regulations concerning digital clinical data. An important issue agreed upon by panellists was that the current ownership model of both data and intellectual property is unclear under the law. Furthermore, panellists agreed that there is a lack of clarity concerning the legal bases for data collection and data sharing. Panellists also agreed that it is currently unclear who holds responsibility for data integrity under law. Other issues agreed upon by panellists included differing data laws between different countries and the unclear regulations concerning international data transfer. Finally, panellists agreed that there is a lack of education concerning data law among all digital surgery stakeholders and poor availability of data law expertise within healthcare facilities.

### Litigation and liability

Although digital surgical systems offer the promise of benefits for patients, surgeons and institutions (Table 1), panellists agreed that there is a lack of regulation concerning litigation and liability, both for failing digital surgical systems and for surgeons who elect to not follow systems such as AI decision support tools. Additionally, if a surgeon were to follow AI decision support, which resulted in a negative outcome, it is unclear how liability would be adjudicated. It is of note, however, that recent guidance published by the American Medical Association concerning AI in healthcare has stated that autonomous AI creators should assume liability^23^. Other issues of importance agreed upon by the panellists included the potential effects of digital surgical systems on medical indemnity and the use of the increased collection of surgical data for the purposes of determining medical negligence.

### Commercial partnerships

The future success of digital surgery is likely to depend upon the development of commercial partnerships who will be able to offer healthcare institutions resources and appropriate expertise. Panellists agreed that business and data sharing models between hospitals and commercial companies were not well defined. Panellists agreed that there is a lack of framework or experience within the majority of institutions for the setting up of fair partnerships between healthcare and commercial entities. They highlighted issues surrounding inequality of power and differing motives between hospitals and commercial companies. Finally, panellists agreed that commercial partnerships may result in restriction on the ability of hospitals to report results.

### Barriers to digital surgery

Panellists were asked to identify key barriers to digital surgery across three areas: development; deployment; and monitoring. The ten consensus barriers identified as most important in previous rounds were ranked during the final online consensus meeting (Table 2).

**Table 2:** Barriers to digital surgery identified and ranked highest to lowest in order of importance by the Delphi panel.

Development	Deployment	Monitoring
Lack of digitisation in hospitals Legacy Hospital IT systems unfit for purpose Insufficient data availability Lack of shared ontology for annotation Lack of data registry and platform standards Lack of standards in data formatting methods Lack of data quality standards Insufficient expertise in surgical AI Poor interoperability between AI systems and embedded technology in the Operating room Difficulties in sharing data between multiple centres	Costs of setting up infrastructure Hindering of process due to bureaucratic processes Challenges in getting contractual relationships established Reimbursement or business model not clearly defined Institutional aversion to sharing patient data Inability to demonstrate safety or clinical benefit to stakeholders Difficulties of integrating AI systems with existing IT infrastructure Variation in hospital IT systems Regulatory requirements are unclear at present Lack of framework for consenting and obtaining data	Clarity on responsibility for data monitoring Lack of resource and personnel dedicated to task Costs associated with monitoring Lack of standardised outcome measures for monitoring Difficulties in quantifying improvement Lack of prioritisation given to monitoring at present Divide between those monitoring and developing surgical AI systems

### Future research goals

Panellists were asked to identify technical, clinical and organisation research goals for future practice. These were subsequently ranked in order of importance during the final consensus meeting (Table 3).

**Table 3:** Future research goals for digital surgery identified and ranked highest to lowest in order of importance by the Delphi panel.

Technical	Clinical	Organisational
Standardisation of surgical data science platforms for data sharing and annotation Shared ontology for data annotation Improving explainability of AI algorithms Dealing with unlabelled or weakly labelled data Identifying inequalities in underlying datasets Effective data collection systems Uptake of common communication standard for surgical data Generation of open source datasets Interoperability between different devices and systems	Define most suitable use cases/applications for surgical AI Develop core outcomes, reporting and measurement sets relevant to AI in surgery Develop framework for introduction and evaluation of AI in surgery Determine trial methodology for assessment of surgical AI Standardisation of processes Encourage surgeons to share data	Demonstrate impact of surgical AI systems Improve public trust and education in AI Legal framework for introduction and monitoring of AI surgical systems Encourage interdisciplinary education Organisation of task force involving all relevant stakeholders to define best practices for surgical AI Define impact of surgical AI systems on litigation and liability Establish a model business plan with industry

### Public response

A total of 20 members of the public answered the scoping questionnaire which was adapted for a non-expert audience. Issues generated from this scoping round were brought forward alongside expert-generated issues to Round 2 for the expert panel to vote upon to ensure the views of the public were appropriately represented. The public panel comprised of a variety of age groups, education levels and self-declared familiarity with AI. Although the public panel had some awareness of digital surgery technologies and the application of surgery to AI, such as robotics, imaging and decision support, there were also common misconceptions around the use of AI replacing human interaction and the extent of autonomy in surgical robotics. The public acknowledged the potential benefits of digital surgical technologies for patients but also for surgical teams with the use of surgical AI as an aid for surgeons a recurring theme.

The public panel were supportive of sharing data for the purposes of surgical AI. However, common themes amongst the public panel were identified concerning the sharing of data including effective cybersecurity, appropriate anonymisation, and understanding who will have access to their data. Concerning transparency and public trust, a common theme amongst the public panel was the need for more knowledge surrounding AI in surgery. Panellists stated that ‘AI is poorly understood by the public’ and that giving access to the public about surgical AI applications would ‘foster trust’. The panel stated that there must be transparency in the presence of adverse outcomes and that failure to disclose this would affect public trust and perceptions.

Finally, the panel had contrasting opinions concerning partnerships between hospitals and commercial companies. Whilst some members of the public understood the value and resources that such partnerships could bring, others were more sceptical, with concerns that companies would sell or profit from their data as well as poor historical records on protecting users’ data. The full results can be found in Supplementary Note 1.

## Discussion

This study is the first in the published literature to define the term digital surgery. Although digital technology is widespread in healthcare, the precise meaning of the term digital surgery is unclear. We present a practical consensus definition which can be utilised by clinicians and academics as well as other stakeholders within digital surgery including patients, industry and policy makers. This builds upon established definitions of terms such as surgical data science which ‘aims to improve the quality of interventional healthcare and its value through the capture, organisation, analysis and modelling of data’^4,5^. While this Delphi exercise demonstrates the potential benefits that digital surgery can bring, we highlight the ethical and data governance issues that developers and utilisers of digital surgical technology will have to contend with. For digital solutions to succeed in the operating room, the ethical and data governance issues identified must not be an afterthought. Instead, it must be at the forefront of those developing and utilising digital surgery applications at all stages from benchtop to bedside.

While many challenges to digital surgery identified by the Delphi panel show parallels to digital health, there is a need to emphasize those which are unique to digital surgery. Firstly, this term is commonly used within the speciality, even though 71% of participants in this analysis agreed that the definition of digital surgery is unclear. If these technologies are to be safely translated into clinical practice and applied in research, then standardisation of this terminology is essential.

Secondly, surgery is a high-risk clinical environment where the consequences of failures in digital technologies have the capacity to cause significant and immediate harm on time scales that are not comparable to other domains of clinical practice. Thirdly, high quality surgical outcomes are dependent on multidisciplinary team performance and behaviours, and therefore the scaling of digital technologies will require broad cultural advances. Fourthly, even the routine recording, and analysis of operating room video data poses unprecedented ethical obstacles that are unique for procedural based specialities. These must be urgently addressed prior to the scaling of these methodologies within operating room environments. This extends beyond patient privacy alone (usually the primary privacy consideration in digital health applications) into the issue of privacy for surgeons and their teams who may be scrutinised for their every action. In this regard, the threat of litigation may serve as a more challenging barrier to the development and adoption of digital surgical tools than in other areas of healthcare. For example, surgeons may be reluctant to allow their data to be used for the development of algorithms, fearing that the same video may be used against them for litigation purposes. Finally, a unique set of barriers exists in accessing potentially large and diverse surgical data sets, which lack standardisation, ontology or quality assurances. Many operating rooms remain steadfastly analogue, and many surgical units lack the technical infrastructure to capture the digital information available to them, or they may even simply choose not to do so. We therefore highlight 3 key areas of focus for digital surgery in the future.

Firstly, digital surgery is here but hospitals and healthcare systems are not ready for it. Significant investment into infrastructure is required if digital surgery is to succeed. Early adopters of digital surgery who have succeeded in setting up this digital infrastructure have had to contend with the dual challenges of bureaucracy and cost. For digital surgery to be adopted at scale, efforts must be made to streamline this process. Template data sharing agreements and commercial models designed specifically for digital surgery applications can act as a starting point for hospitals engaging in complex and time-consuming negotiations. Appropriate commercial and legal expertise must however be made available for tailored advice. The UK has set up a National Centre of Expertise to oversee and provide guidance for hospitals engaging in these partnerships. Success may also be found in the development of a national health research data hub in surgery in a similar fashion to established national health research data hubs in areas such as pain, mental health and cancer care^24^.

Panellists identified interoperability as a key issue in incorporating digital surgery into healthcare. Surgical data standards must be defined and steps towards this have already been taken with the recent publication of data annotation standards^25^. The challenges of interoperability extend beyond digital surgery and pose issues for the broader application of digital technology within healthcare. Current issues with data sharing between devices or hospitals will be complicated further with future applications requiring global data pooling. International data sharing processes will have to contend with interoperability issues on a backdrop of evolving privacy requirements and future efforts must aim to standardise and streamline this process.

Modern-day operating theatres have the potential to generate extensive and heterogenous datasets but the majority of hospitals, at present, fail to capitalise on this. Hospitals lack the technical storage, network and cybersecurity capabilities and funding required to maintain pace with advances in technology. Digital surgery must also contend with broader issues across digital health such as varying levels of digitisation across public and private hospitals or national providers, coupled with the problems of heterogenous hospital IT systems and electronic health records, all of which create considerable barriers for the adoption of safe digital surgery technologies at scale.

Lastly, although not specific to digital surgery, issues of cybersecurity must not be overlooked. Digital surgical systems operate in high-risk clinical environments and breaches in cybersecurity affecting them have the potential to cause significantly more patient harm compared to other medical specialties. Although no patient harm was reported from the 2017 WannaCry malware attack, it highlighted the vulnerability of hospitals to cybersecurity threats^26,27^. Cybersecurity measures must not only be suitably robust to protect these systems but cater for ‘worst but possible’ scenarios.

Secondly, public and patient involvement is vital for development and deployment of digital surgery. Our public panel has shown that patients are supportive of digital surgery and willing to donate data. Concerns arose mainly with lack of awareness with what digital surgery entails and how patient data will be utilised. This lack of awareness surrounding digital surgery may result in poor understanding of the benefits available from digital surgery compared to current surgical practice.

Transparency and public trust are consistently highlighted as key issues across both our public and expert panels and across guidance into AI in other fields. The public are a key stakeholder within digital surgery and involvement of the public at all stages of development and deployment is vital to foster trust. We must not forget that patients are at the heart of digital surgery. Public acceptability of digital surgery applications and the collection and sharing of data that they may require must not be overlooked.

However, our public panel has shown that levels of understanding of digital surgery and AI vary significantly. Patients may not fully understand the extent of data collection, how it may affect them or what digital surgery entails. Patient education concerning digital surgery can build upon existing initiatives such as the Wellcome Trust’s ‘Understanding patient data’ programme^28^ and allow patients to be educated about what data is collected and how it is utilised in digital surgery applications. It is only through educating and engaging with the public that they can provide suitably informed consent as to whether they want to share their data.

Thirdly, education is not only required for patients but for all stakeholders. While an important research goal identified by our panel was the need to determine the most suitable applications for surgical AI, this will only be achieved if there is sufficient interdisciplinary education. Technologists must have an awareness of the surgical challenges that digital technology may be able to solve. Similarly, clinicians must understand the basis of the technologies they are using if they are to be advocates for their patients.

Furthermore, this Delphi exercise has revealed the wider ethical, data governance and legal issues surrounding digital surgery. Panellists identified that there is poor understanding concerning the legal issues as well as the lack of legal expertise within hospitals. Efforts must be made to educate stakeholders and seek expertise around these issues and for them to be aware of the evolving legal and regulatory landscape which may extend beyond data privacy law and include issues of competition law and intellectual property protection rights, as well as commercial considerations such as liabilities, indemnities and data ownership. The future digital surgeon will not only be a surgeon; they must have an understanding of AI and technology as well an awareness around the legal, ethical and data governance issues concerning their use.

While Delphi methodology has been successfully employed in the literature to provide consensus opinion^29,30^, it has limitations. Firstly, the conclusions drawn from the Delphi exercise are the subjective opinion of a single group. To mitigate for this effect, efforts were made to reduce bias and ensure the conclusions drawn were representative by recruiting a large number of experts with national or international profiles from a range of key fields within digital surgery. Moreover, in areas such as digital surgery where there is limited existing knowledge and a need for knowledge to be drawn upon from multiple different areas of expertise, Delphi exercises have been shown to be a highly effective methodology^31^.

Secondly, the reliability of Delphi methodology has been criticised due to a lack of methodological standardisation^20^. We aimed to improve the reliability of this study by drawing upon existing methodology used within the literature. We also addressed this limitation through extensive discussion in the final consensus meeting to ensure conclusions drawn were valid and appropriate.

Finally, it could be argued that our consensus definition of ‘digital surgery’ may lack specificity, for example by failing to expand on the terms such as ‘technology’. This was widely debated by the Delphi panel and the final consensus definition was agreed upon for several reasons. Firstly, it is unclear how to prioritise technologies which should or should not be incorporated within the definition and erroneous conclusions could be drawn from technologies that have been omitted. Secondly, by listing all technologies which should be incorporated, the definition would be significantly lengthened, limiting its practical use. Finally, the definition would lack future proofing; by strictly defining the technologies included within digital surgery, technologies which are not currently developed are excluded. We therefore believe that this first consensus definition of ‘digital surgery’ satisfies the aim of creating a usable definition and may act as a platform for future iterations.

In conclusion, this Delphi exercise defines digital surgery as the use of technology for the enhancement of preoperative planning, surgical performance, therapeutic support, or training, to improve outcomes and reduce harm. Data generating technologies present both opportunities and risks. This Delphi has identified key ethical issues, barriers and research goals which will serve as a foundation to steer future research in this area. Issues surrounding data, privacy, confidentiality and public trust, consent, law, litigation and liability, and commercial partnerships must be considered at all stages by those developing and utilising digital surgery. Future research into the issues identified must involve all digital surgery stakeholders, and therefore work in partnership with patients.

## Methods

**Fig. 2:**
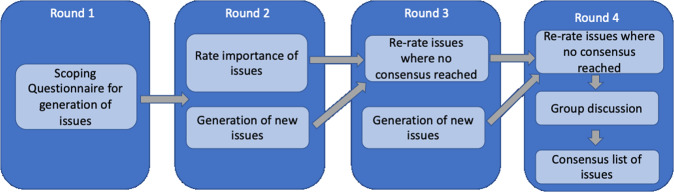
Structure of Delphi exercise^32^. Round 1 consisted of a scoping questionnaire. Rounds 2 and 3 consisted of voting questionnaires. Round 4 consisted of a final online meeting.

The protocol for this Delphi consensus study has previously been published^32^. The structure of the Delphi exercise consisted of four rounds (Fig. 2). Round 1 consisted of an initial scoping round, which invited panellists to generate issues around themes identified from the literature. In Rounds 2 and 3, experts voted on the issues generated in Round 1 with respect to importance or agreement. Round 4 consisted of a final online consensus meeting amongst panellists firstly to vote on non-consensus statements and secondly to discuss consensus statements from the previous rounds.

### Expert panel

Experts from the fields of surgery, AI, industry, law, ethics and policy were invited to participate. All invited participants had national or international profiles in their respected fields and/or were authors of high impact research in this area. 122 participants were initially approached via email to express their interest in participating in the Delphi exercise. Of the 38 participants that completed all rounds of the Delphi exercise, 24 were surgeons with an interest in digital technology, 8 were academics with expertise in AI and its application to surgery, 3 were from healthcare industry with the remaining participants involved in the fields of healthcare policy, digital law and ethics. 18 participants were from the UK, 13 participants from the rest of Europe, 6 participants from North America and 1 participant from South America. The median (range) h-index for the participants was 26 (5–76) and the participants had a median (range) of 15 (4–32) years experience.

### Public panel

Members of the public, as key stakeholders, were invited to participate in Round 1 of the Delphi exercise. A simplified version of the Round 1 questionnaire presented to the expert panel was adapted for a non-expert audience (see Supplementary Note 1). There were no qualifying criteria or prior knowledge required for participation. The public panel were recruited through the VOICE platform (https://www.voice-global.org/), an organisation which comprises of members of the public across the world who volunteer to contribute their insights to health research.

### Round 1

A review of the literature surrounding data governance and ethical issues across the implementation of digital surgery identified key themes which formed the basis of the scoping round^13,33–37^. In addition, participants were asked about their understanding of the term digital surgery and to identify key barriers and future research goals concerning digital surgery (see Supplementary Methods for full questionnaire). The purpose of this initial scoping round was to encourage generation of issues across these themes.

### Round 2

Issues generated both by the expert panel and the layperson participants in Round 1 were thematically analysed using NVivo qualitative data analysis software (QSR International Pty Ltd. Version 12, 2018) in order to generate statements for Round 2. Statements generated from Round 1 in addition to the public panel responses were then presented to the expert panel through Qualtrics XM platform (Qualtrics, Provo, UT). Panellists were asked to rate statements on a 9-point Likert scale according to either importance or agreement. Consensus was defined a priori if the issue was deemed between 7–9 (important to totally important) by at least 70% of the panel and between 1–3 (totally unimportant to unimportant) by fewer than 30% of the panel, a popular approach used in Delphi exercises^38^. Panellists were also encouraged to suggest additional statements or modifications to the statements.

### Round 3

Statements failing to reach consensus in Round 2 in addition to novel statements generated in Round 2 were presented to panellists in Round 3. Results from the previous round alongside summary statistics were presented to all panellists in order to encourage convergence of opinion for non-consensus statements. Panellists voted on statements in a similar manner to Round 2.

### Round 4

Panellists who had completed all previous rounds of the Delphi exercise were invited to a final consensus meeting held on the Microsoft Teams (Microsoft, Redmond, WA) platform on the 24/06/2021. Statements failing to reach consensus from Round 3 were discussed and subsequently voted upon during the meeting using Mentimeter (Mentimeter, Stockholm, Sweden), a real-time polling software. During this final consensus meeting, statements were discussed amongst panellists in order to draught a consensus document. Finally, barriers to the development, deployment and monitoring of digital surgery and future research goals which had reached consensus in the previous rounds were ranked from most to least important by meeting participants.

### Ethical approval

Ethical approval for this study was granted by the local research ethics committee at Imperial College, London (20IC6136). All participants were electronically provided with participant information prior to commencing Round 1. All participants provided electronic informed consent prior to commencing Round 1.

### Reporting summary

Further information on research design is available in the Nature Research Reporting Summary linked to this article.

## Supplementary information

Supplementary information

Reporting Summary Checklist

## Data Availability

The datasets generated during the current study are available from the corresponding author on reasonable request.
