# Supplementary material for: A Delphi consensus statement for digital surgery
Source: NPJ Digit Med. 2022 Jul 19;5:100. doi: 10.1038/s41746-022-00641-6 (PMC9296639; doi:10.1038/s41746-022-00641-6)
Supplement: Supplementary file 1 — Supplementary information [file 41746_2022_641_MOESM1_ESM.pdf]

SUPPLEMENTARY INFORMATION

Supplement Methods: Round 1 questionnaire ii

Supplement Note 1: Round 1 Public questionnaire and results iii

Supplement Note 2: Round 2 results viii

Supplement Note 3: Round 3 results xix

Supplement Note 4: List of consensus statements xxiii

Supplement Methods: Round 1 questionnaire

1. What is your understanding of the term ‘digital Surgery’?
2. What are the key components of digital surgery or AI in surgery?
3. What do you think are the key benefits of using AI in surgery?
4. What are the key issues concerning data access in AI and surgery?
5. What are the key issues concerning data sharing and ownership in AI and surgery?
6. What are the key issues concerning data storage and security in AI and surgery?
7. What are the key issues concerning data law in AI and surgery?
8. What are the key issues concerning privacy and confidentiality in AI and surgery both for patients and surgical teams?
9. What are the key issues concerning consent in AI and surgery?
10. What are the key issues concerning litigation and liability in AI and surgery?
11. What are the key issues concerning transparency and public trust in AI and surgery?
12. What are the key issues concerning commercial partnerships in AI and surgery?
13. What are the technical and organisational barriers to developing AI surgical systems?
14. What are the technical and organisational barriers to deploying AI surgical systems?
15. What are the technical and organisational barriers to monitoring AI surgical systems?
16. What are the key future research goals for the implementation of AI in surgery?

Supplementary Note 1: Round 1 Public questionnaire and results

Demographics

**Sex**	**Number**
Female	13
Male	7

**Age**	**Number**
18-24	2
25-34	3
35-44	1
45-54	1
55-64	3
65-74	8
75+	2

**Highest level of qualification**	**Number**
GCSE/O-level	2
College	2
Bachelors	9
Masters	4
Doctorate	3

**Self-declared familiarity with AI**	**Number**
No knowledge	0
Poor	3
Average	14
Good	3
Very good	0

The responses of the public were thematically analysed and a representation of the responses as categorised by their self-declared familiarity with AI is shown below.

What do you understand by the term ‘artificial intelligence’ (AI) and specifically AI in surgery?

Poor: ‘computer based brain which can make decisions based on its programming’ ‘operating tools and procedures which replace human use’

Ave: ‘decision making’ ‘supportive activities’ ‘repetitive tasks’ ‘use of previous data to input into current surgical robotics’

Good: ‘evaluate data without human intervention’ ‘make decisions based on large quantities of prior surgical data’ ‘Machines are taught a procedure and learn in training to get better at carrying out the task’ ‘computerising means of surgery’

What types of technology do you think surgical AI involves?

Poor: ‘camera can see the site of the operation’ ‘able to control an instrument remotely’

Ave: ‘Robotic surgery’ ‘a lot of it will be in scans, imaging, perhaps with some analysis to help decisions’ ‘back up support for surgeons’ ‘less invasive intervention’ ‘more consistent results’ ‘less strain on the surgeon’

Good: ‘Data handling’ ‘Significant computing power’ ‘Precision movement machinery’

At this stage, participants were shown videos of applications of AI technology in surgery and a short written explanation was given.

What do you think is the usefulness or benefits of surgical AI?

Poor: ‘Robots doing operations? I am nervous, but only in the same way that I am with driverless cars’ ‘[faster] speed of operation benefits the surgeon and patient’ ‘gaining more detailed information’ ‘not relying on human movements or thoughts which could be affected by fatigue/nerves’

Ave: ‘Surgery carried out to a consistent minimum standard’ ‘Reduce errors’ ‘Aid decision making and carrying out of surgery’ ‘Best use of resources’ ‘it can do things people cannot and can enhance all surgical experiences’

Good: ‘Data driven results’ ‘Surgeon aids’ Obtain masses of data for future comparison’

What do you think are the issues around using data to create or use AI in surgery? *This data can range from personalised data such as your age, gender and past medical history to non-identifiable information such as a video of your operation or how efficiently the surgeon uses their instruments.*

Poor: ‘You should use all the data you can to make it safe’ ‘People may be concerned that they would be able to be identified’

Ave: ‘it needs to be good quality/non biased’ ‘data storage can be hacked and information removed’ ‘there would be no issues for me if it was all non-identifiable’ ‘consultants/administrators/IT managers all tend to act as gatekeepers for patient’s data. I absolutely want my data shared. Facebook/Morrison’s all keep more data on me than the NHS’ ‘Sell my data and use the money to fund further research’

Good: ‘Sheer amount of data to be stored’ ‘Problems in who has security access to data’ ‘Safe storage of data against cyberattack’ ‘There are no issues. The issues in my opinion are irrelevant religious and moral medieval objections’

What do you think the issues are surrounding privacy and confidentiality when using AI in surgery?

Poor: ‘I would personally have no problem, as I feel anything I can contribute to the learning of scientists, Drs etc is of benefit’ ‘I don't think they are any different from the issues when using conventional surgery’ ‘as long as people can be assured on anonymity that I think that it would be fine.’

Ave: ‘Hacking but that is a problem everywhere we have to live with it’ ‘Don’t know what issues there could be’ ‘Videos are a particular concern as risk patient could be identifiable in a vulnerable state’

Good: ‘large amounts of data must be handled’ ‘avoid individuals being identified’ ‘needs ethical framework’ ‘patients need to understand and agree the use of data under current privacy regulations’

What do you think the issues are around consent when using AI in surgery?

Poor: ‘I personally would be happy to consent as long as it was a tried and tested part of the surgery, that the medical professionals were experienced in it’ ‘The more that AI is used in surgery, the more training professionals receive and the more familiar people become with it should make consent easier.’

Ave: ‘Does the patient know what they are consenting to’ ‘There are none. You either take the risk of getting better or would you rather worry about protecting your data’ ‘Will surgeons be too involved with machinery to focus’ ‘Lack of understanding what is involved in technology so making it difficult to give informed consent’

Good: ‘Need individuals to consent to data being used and individuals need a good understanding of what those data include’ ‘fears which the patient has about this technology. There is much fake information out there which may have influenced them’

What do you think are the issues around transparency and public trust when using AI in surgery?

Poor: 'It's the same as exploring a Dr's records, it's almost impossible. AI might be more transparent' 'the more people know about it the better, it needs more media involvement, seeing surgeries on TV. Medical professionals should communicate to public and build trust'

Ave: 'give access to what is being done by AI in surgery to foster public trust' 'technology not understood by many people leading to distrust' 'Good honest relationships between patient and surgeons needed' 'lack of transparency if things go wrong. This would affect public trust and perception'

Good: 'AI is poorly understood by the public. Would need to make it very clear what the imposed limitations in decision making of AI are' 'explain to the public without getting technical' 'risk assessment needed rather than a list of what could go wrong'

What do you think are the issues around working with commercial companies when developing or using AI systems in surgery? *Some examples of these companies include Microsoft or Google who work with hospitals in order to develop AI tools.*

Poor: 'Would they make a profit out of my data?' 'Commercial companies have got to make a profit' 'if it is being done to a budget, will corners be cut'

Ave: 'Profit will be prioritised over healthcare. Company should not be allowed to use the data for other purposes' 'using the data for commercial purposes' 'commercial companies may wish to keep their patients and not share advances that would improve life and welfare not just those who can pay' 'Google knows more about me than I do. That is how things are these days' ' I welcome it. They have superior systems to the NHS and will advance stuff in a year which normally take the NHS 10 years'

Good: 'large commercial companies have a very poor record on protecting users' data' 'They have the developmental resources and skills. Of course they should be involved'

What do you think are the barriers to developing and using surgical AI systems? *Here, we're interested in any suggestions and this can range from technological aspects to regulatory aspects, so please share your thoughts!*

Poor: 'Cost will be enormous' 'Do the hospitals have the basic infrastructure to support this level of technology' 'Are we supporting medical AI careers'

Ave: 'Only wealth institutes or those working with commercial companies can afford AI' 'Laws take time and can't keep up with AI advances' 'increase global inequality' 'will it deskill surgeons' 'public trust may be a barrier initially' 'evidence of benefit or not'

Good: 'lack of high quality/quantity data' 'public trust in AI' 'clinician trust in AI' 'reliability of AI must be very high'

What do you think future research into surgical AI should focus on? *Here, we want to know what you think our key priorities should be.*

Poor: ‘simple operations that could be carried out by robots’ ‘using AI tools and equipment in areas where people are unable/unsafe to be’ ‘planning reconstructive surgery’

Ave: ‘affordability and mobility – getting AI surgery to most people’ ‘improving diagnoses’ ‘less stress on surgeons’ ‘public education’ ‘routine or resource intensive tasks to free up time’

Good: ‘maintain safety standards on AI processes’ ‘AI to assist the surgeon’ ‘AI for one task to ease a multitask operation’

Supplement Note 2: Round 2 results

1. To what extent do you agree/disagree with the following statements about digital surgery

	Median (IQR)	1-3	4-6	7-9	Prefer not to answer	Consensus
The definition of digital surgery is unclear	7 (6-8)	3	14	27	0	No
Digital surgery concerns pre-operative, peri-operative and post-operative aspects of surgery	8 (7-9)	1	4	38	1	Yes
Digital surgery is not restricted to the operating theatre but incorporates other aspects of surgery such as training, diagnosis and investigation	9 (8-9)	0	6	36	2	Yes

2. To what extent do you agree/disagree that the following are components of digital surgery

	Median (IQR)	1-3	4-6	7-9	Prefer not to answer	Consensus
Artificial intelligence	9 (8-9)	0	3	41	0	Yes
Augmented or virtual reality	9 (8-9)	0	2	42	0	Yes
Cloud computing	8 (6-9)	1	13	30	0	No
Computer vision	9 (8-9)	0	1	43	0	Yes
Data capture	9 (8-9)	0	3	41	0	Yes
Data annotation	9 (7.75-9)	0	5	39	0	Yes
Data storage	8 (6-9)	1	11	32	0	Yes
Data analysis	9 (7.75-9)	0	6	38	0	Yes
Digital patient pathways	8 (7-9)	0	7	37	0	Yes
Electronic health records	8 (7-9)	2	6	36	0	Yes
Advanced imaging systems	9 (8-9)	1	3	40	0	Yes
Machine learning	9 (8-9)	0	1	43	0	Yes
Robotics	8.5 (7-9)	1	6	37	0	Yes
Surgical navigation systems	9 (8-9)	0	3	41	0	Yes
Remote monitoring	8 (7-9)	1	8	35	0	Yes
Sensor technology	9 (8-9)	1	5	38	0	Yes
Telesurgery	8.5 (7.75-9)	0	4	40	0	Yes

3. To what extent do you agree/disagree that the following are benefits of digital surgery

	**Median (IQR)**	**1-3**	**4-6**	**7-9**	**Prefer not to answer**	**Consensus**
Improving diagnostics	8 (7-9)	2	4	38	0	Yes
Improving surgical efficiency	8 (8-9)	0	0	44	0	Yes
Improving clinical outcomes	8.5 (8-9)	0	0	44	0	Yes
Improving cost effectiveness	8 (7-9)	1	8	35	0	Yes
Improving patient care	8.5 (8-9)	0	2	42	0	Yes
Delivering patient specific treatment	8 (8-9)	0	3	41	0	Yes
Allowing pre-operative treatment planning	8 (8-9)	0	4	40	0	Yes
Identifying patient deterioration more promptly (an 'early warning system' for surgeons)	8 (7-9)	1	3	39	1	Yes
Error prediction	9 (8-9)	0	3	41	0	Yes
Error detection	9 (8-9)	0	4	40	0	Yes
Automation of surgical processes	8 (7-9)	2	6	36	0	Yes
Providing decision support to the surgeon	9 (8-9)	0	2	42	0	Yes
Reducing cognitive load on the surgeon	8 (7-9)	2	6	36	0	Yes
Standardisation of surgical processes	8 (8-9)	0	2	42	0	Yes
Evaluating surgeon performance	8 (7-9)	1	3	40	0	Yes
Improving surgeon ergonomics and health	8 (7-9)	1	9	34	0	Yes
Accelerating surgical education	8 (7-9)	1	3	40	0	Yes

4. Please rate how important you consider the following issues concerning data access and AI in surgery

	Median (IQR)	1-3	4-6	7-9	Prefer not to answer	Consensus
Lack of infrastructure for data acquisition	8 (8-9)	0	1	43	0	Yes
Data is not readily available in a digital format	8 (7-9)	0	3	41	0	Yes
Lack of data quality standards	8 (8-9)	0	1	43	0	Yes
Lack of data annotation standards	8 (7.75-9)	0	7	37	0	Yes
Lack of data formatting standards	8 (7-8)	0	9	35	0	Yes
Determining appropriate access to data	8 (7-9)	0	4	39	1	Yes
Governance processes at present are overcomplicated and obstructive	8 (7-9)	0	4	40	0	Yes
Lack of reliable datasets	8 (8-9)	0	4	39	1	Yes
Lack of interoperability between different devices and systems	9 (8-9)	0	3	40	1	Yes

5. Please rate how important you consider the following issues concerning data storage and security and AI in surgery

	Median (IQR)	1-3	4-6	7-9	Prefer not to answer	Consensus
Appropriate encryption	9 (7.25-9)	0	2	40	2	Yes
Lack of structure in data storage	8 (7-8)	0	6	37	1	Yes
Hospitals currently lack the technical ability for data storage	8 (7-9)	0	8	36	0	Yes
Costs of data storage	6 (6-8)	5	18	21	0	No
Institutions are not equipped and under resourced to perform appropriate cybersecurity	8 (7-8)	2	5	37	0	Yes
Implications of data breaches are poorly defined	8 (7-8)	0	9	34	1	Yes

6. Please rate how important you consider the following issues concerning data sharing and AI in surgery

	Median (IQR)	1-3	4-6	7-9	Prefer not to answer	Consensus
No guidelines concerning ownership of data	8 (7-9)	1	5	38	0	Yes
Adherence to present data rules can hamper competitiveness	7 (6-8)	2	13	28	1	No
No consensus on data sharing formats	8 (7-8)	0	8	36	0	Yes
Data sharing across international boundaries is problematic	8 (7-9)	0	9	35	0	Yes
Legal requirements concerning data sharing are unclear	8 (7-9)	2	6	36	0	Yes
Lack of motives for surgeons to share data	7 (5.75-8)	0	15	29	0	No

7. Please rate how important you consider the following issues concerning privacy and confidentiality and AI in surgery

	Median (IQR)	1-3	4-6	7-9	Prefer not to answer	Consensus
Appropriate anonymisation of data	9 (8-9)	1	1	42	0	Yes
Determining patient agreements for data sharing	9 (8-9)	0	3	41	0	Yes
Ensuring public trust on data sharing	9 (8-9)	0	3	41	0	Yes
Ensuring data is not used for other purposes if held	8 (7-9)	1	8	35	0	Yes
Lack of education among surgical teams about the significance of data they are collecting	8 (7-9)	1	7	36	0	Yes
The surgeon's right to privacy and influence on behaviour within the Operating Room	8 (6-9)	1	12	31	0	Yes

8a. Please rate how important you consider the following issues concerning consent and AI in surgery

	Median (IQR)	1-3	4-6	7-9	Prefer not to answer	Consensus
Difficulties with consent if we do not know the future applications	8 (7-8.25)	0	9	35	0	Yes
Incentivising patients to share data	7 (6-8)	1	11	32	0	Yes
Management of a patient who chooses to withdraw consent	8 (6-8)	0	12	32	0	Yes
Educating patients so consent is more informed	8 (6.75-8)	1	10	33	0	Yes
Ensuring patients fully understand what is being asked	8 (7-9)	1	7	36	0	Yes
Differing requirements of consent between countries	8 (6-9)	2	11	31	0	No
Rights of the surgeon and the wider surgical team to opt out	8 (7-9)	2	6	36	0	Yes

8b. To what extent do you agree/disagree with the following statements about the consent process for surgical AI applications

	Median (IQR)	1-3	4-6	7-9	Prefer not to answer	Consensus
Consent should incorporate not only the patient but also the wider surgical team	7.5 (6-8)	5	11	28	0	No
Consent procedures should delineate the extent of data collection	8 (7-8)	0	5	39	0	Yes
Consent procedures should delineate who will access the data	8 (7-9)	0	9	35	0	Yes
Consent procedures should explain why the data will be collected	8 (7-9)	0	2	42	0	Yes
Consent procedures should allow data collection for future or unknown applications	8 (7-9)	1	8	35	0	Yes
Patients should be consented separately should commercial partners have access to their data	8 (6-8.25)	4	9	31	0	Yes
There should be a standardised methodology for consenting patients to share their data	8 (7-9)	1	3	40	0	Yes

9. Please rate how important you consider the following issues concerning transparency and public trust and AI in surgery

	Median (IQR)	1-3	4-6	7-9	Prefer not to answer	Consensus
Lack of explainability due to opaqueness of surgical AI systems	7 (7-8)	0	8	36	0	Yes
Failure to produce an effective system to date	7 (6-8)	5	9	30	0	No
Mandatory reporting of outcomes regardless of success/failure	8 (7-9)	0	9	35	0	Yes
Lack of engagement with the public to date	7 (6-8)	3	11	30	0	No
Lack of education around AI among the public	8 (6-8)	3	10	31	0	Yes
Lack of existence of neutral body looking at public trust in AI	7 (6-8)	2	12	30	0	No
Fear of AI reinforcing biases in datasets	8 (6-8)	1	13	30	0	No

10. Please rate how important you consider the following issues concerning the law and AI in surgery

	Median (IQR)	1-3	4-6	7-9	Prefer not to answer	Consensus
Lack of standardisation of terminology concerning AI in law	7 (6-8)	0	14	30	0	No
Law adapting behind technology curve and not fit for purpose	7 (6-8.25)	2	10	32	0	Yes
Lack of dedicated regulations concerning digital clinical data	7 (7-8)	0	8	36	0	Yes
Legal bases for data collection are unclear	7 (6-8)	1	11	32	0	Yes
Unclear who holds responsibility for data integrity under law	7 (7-8)	0	9	35	0	Yes
Data ownership model is unclear under the law	7 (7-8)	0	9	35	0	Yes
Lack of clarity legally concerning permissions to share data	7 (6-8)	2	11	31	0	Yes
Legal rights to privacy for the surgeon unknown	7 (6-8)	4	9	31	0	Yes
Regulations concerning international data transfer unclear	8 (5.75-8)	4	11	29	0	No
Differing data laws between different countries	7 (6-8)	4	11	29	0	No
Lack of education among stakeholders concerning data law	8 (7-8)	0	8	36	0	Yes
Poor availability of data law expertise within healthcare facilities	8 (7-8.25)	1	9	34	0	Yes
Ownership of intellectual property under law	7 (7-8)	1	9	33	1	Yes

11. Please rate how important you consider the following issues concerning litigation and liability and AI in surgery

	Median (IQR)	1-3	4-6	7-9	Prefer not to answer	Consensus
Determining lines of responsibility – who is responsible if AI fails?	9 (8-9)	0	1	43	0	Yes
Determining liability if surgeons do not follow AI decision support tools	8 (7-9)	1	2	41	0	Yes
Effects of surgical AI on medical indemnity/insurance	8 (7-9)	1	4	38	1	Yes
Consequences of increased recording of data and its potential future use to determine medical negligence	8 (7-9)	0	8	35	1	Yes
Lack of regulations, at present, concerning litigation and liability	8 (7-9)	0	5	39	0	Yes

12. Please rate how important you consider the following issues concerning commercial partnerships and AI in surgery

	Median (IQR)	1-3	4-6	7-9	Prefer not to answer	Consensus
Business model between hospitals and commercial companies not defined	8 (7-9)	1	7	36	0	Yes
Lack of framework or experience within the majority of institutions for the setting up of fair partnerships	7 (7-9)	0	6	38	0	Yes
Data sharing protocols not defined	8 (7-9)	0	7	37	0	Yes
Differing motives between hospitals and commercial companies	7 (7-8)	3	7	34	0	Yes
Handling inequality of power between involved parties	7 (6-8)	1	17	26	0	No
Patients may be less inclined to give data if commercial companies involved	7 (6-8)	1	15	28	0	No
Restriction on physician autonomy	7 (5-8)	2	18	24	0	No
Restriction on hospitals to report results	7 (6-8)	1	12	31	0	Yes
Vendor lock-in	7 (5-8)	2	15	25	2	No
Defining force majeure (hospital/industry fails to meet contractual obligations due to unforeseen circumstances)	7 (5-8)	1	19	22	2	No

13. Please rate how important you consider the following barriers to developing AI in surgery

	Median (IQR)	1-3	4-6	7-9	Prefer not to answer	Consensus
Lack of digitisation in hospitals	8 (7.75-9)	2	0	42	0	Yes
Hospital IT systems not fit for purpose	8 (8-9)	1	1	42	0	Yes
Insufficient data availability	8 (7-8)	1	1	42	0	Yes
Lack of shared ontology for annotation	8 (7-8)	0	9	35	0	Yes
Burden of annotation	8 (7-9)	1	5	38	0	Yes
Lack of data registry and platform standards	8 (7-9)	1	2	41	0	Yes
Lack of standards in data formatting methods	8 (7-8.25)	0	4	40	0	Yes
Lack of data quality standards	8 (7-9)	0	5	39	0	Yes
Insufficient expertise in surgical AI	8 (7-9)	1	9	34	0	Yes
Identification of the right problems to solve	8 (6.75-9)	2	9	33	0	Yes
Poor interoperability between AI systems and embedded technology in the OR	8 (7-9)	0	6	38	0	Yes
Difficulties in sharing data between multiple centres	8 (7-8)	1	6	37	0	Yes
Lack of international collaboration	7 (5.75-8)	2	13	29	0	No
Lack of funding into surgical AI projects	7 (6-9)	4	11	29	0	No
No success stories so far to drive field	8 (6-9)	2	10	32	0	Yes
Priorities of clinicians and AI scientists not aligned	7.5 (6.75-8)	2	9	33	0	Yes
Lack of knowledge of how to perform trials which demonstrate safety and utility of AI systems	8 (6.75-9)	3	8	33	0	Yes

14. Please rate how important you consider the following barriers to deploying AI in surgery

	Median (IQR)	1-3	4-6	7-9	Prefer not to answer	Consensus
Costs of setting up infrastructure	8 (7-9)	2	7	35	0	Yes
Medical workforce resistance	7 (6-7)	1	13	30	0	No
Resistance from hospital management	7 (6-8)	1	15	28	0	No
Hindering of process due to bureaucratic processes	8 (7-8)	1	8	35	0	Yes
Challenges in getting contractual relationships established	7.5 (7-8.25)	1	9	34	0	Yes
Reimbursement or business model not clearly defined	7 (6.75-8)	1	10	33	0	Yes
Lack of prioritisation given to new technology	7 (6-8)	2	11	31	0	Yes
Institutional aversion to sharing patient data	7.5 (6.75-9)	1	10	33	0	Yes
Hesitancy concerning cloud technologies	7 (5.75-8)	4	15	25	0	No
Inability to demonstrate safety or clinical benefit to stakeholders	8 (7-9)	1	8	35	0	Yes
Insufficient cybersecurity measures	7.5 (6-8)	2	13	29	0	No
Difficulties of integrating AI systems with existing IT infrastructure	8 (7-9)	2	7	35	0	Yes
Network connectivity issues	7 (6-8.25)	5	10	29	0	No
Variation in hospital IT systems	8 (6-9)	2	10	32	0	Yes
Regulatory requirements are unclear at present	8 (7-9)	1	8	35	0	Yes
Lack of framework for consenting and obtaining data	8 (7-9)	1	6	37	0	Yes
Lack of AI literacy among surgical teams	7 (6-8.25)	2	11	31	0	Yes
Lack of public trust in AI	7 (6-8)	1	18	25	0	No

15. Please rate how important you consider the following barriers to monitoring AI in surgery

	Median (IQR)	1-3	4-6	7-9	Prefer not to answer	Consensus
Unclear who should be responsible for monitoring	7.5 (7-9)	0	10	34	0	Yes
Lack of resource and personnel dedicated to task	7 (6-8.25)	1	14	29	0	No
Costs associated with monitoring	7 (6-9)	1	14	29	0	No
Lack of standardised outcome measures for monitoring	8 (7-9)	1	8	35	0	Yes
Difficulties in quantifying improvement	8 (6.75-9)	0	11	33	0	Yes
Lack of prioritisation given to monitoring at present	8 (6-8)	0	12	32	0	Yes
No capability for remote monitoring at present	7 (6-8)	2	18	24	0	No
Divide between those monitoring and developing surgical AI systems	7 (6-8)	5	11	28	0	No

16. To what extent do you agree/disagree with these technical future research goals for surgery in AI

	Median (IQR)	1-3	4-6	7-9	Prefer not to answer	Consensus
Standardisation of surgical data science platforms for data sharing and annotation	8 (7-9)	0	5	39	0	Yes
Shared ontology for data annotation	8 (7-9)	0	5	39	0	Yes
Dealing with unlabeled or weakly labelled data	8 (7-8.25)	2	8	34	0	Yes
Uptake of common communication standard for surgical data	8 (7-9)	0	8	36	0	Yes
Effective data collection systems	8 (7-9)	1	4	39	0	Yes
Generation of open-source datasets	8 (7-9)	2	6	36	0	Yes
Interoperability between different devices and systems	8 (7-9)	0	2	42	0	Yes
Improving explainability of AI algorithms	8 (7-9)	1	5	38	0	Yes
Identifying inequalities in underlying datasets	8 (7-9)	0	9	34	1	Yes

17. To what extent do you agree/disagree with these clinical future research goals for surgery in AI

	**Median (IQR)**	**1-3**	**4-6**	**7-9**	**Prefer not to answer**	**Consensus**
Define most suitable use cases/applications for surgical AI	9 (8-9)	0	2	42	0	Yes
Develop core outcomes, reporting and measurement sets relevant to AI in surgery	8 (7-9)	1	2	41	0	Yes
Develop framework for introduction and evaluation of AI in surgery	8.5 (7.75-9)	1	4	39	0	Yes
Determine trial methodology for assessment of surgical AI	8 (7-9)	0	5	39	0	Yes
Encourage surgeons to share data	8 (7-9)	1	7	36	0	Yes
Standardisation of processes	8 (7-9)	0	4	40	0	Yes

18. To what extent do you agree/disagree with these organisational future research goals for surgery in AI

	**Median (IQR)**	**1-3**	**4-6**	**7-9**	**Prefer not to answer**	**Consensus**
Demonstrate impact of surgical AI systems	9 (8-9)	0	0	44	0	Yes
Establish a model business plan with industry	7 (6.75-8)	1	10	33	0	Yes
Improve public trust and education in AI	7.5 (7-9)	0	7	37	0	Yes
Encourage interdisciplinary education – clinicians should understand basic AI, AI scientists should understand clinical needs	8 (7-9)	1	4	39	0	Yes
Legal framework for introduction and monitoring of AI surgical systems	8 (7-9)	1	6	37	0	Yes
Define impact of surgical AI systems on litigation and liability	8 (7-9)	1	8	34	1	Yes
Organisation of task force involving all relevant stakeholders to define best practice standards for surgical AI	8 (7-9)	1	6	37	0	Yes

Supplement Note 3: Round 3 results

1. To what extent do you agree/disagree with the following statements about digital surgery

	Median (IQR)	1-3	4-6	7-9	Prefer not to answer	Consensus
The definition of digital surgery is unclear	7 (6-8)	4	7	27	0	Yes
NEW: Novel definition of digital surgery: “Digital surgery can be defined as the data-driven enhancement of surgical diagnosis, therapeutic decision support and training by means of digital technology including information processing, artificial intelligence and robotics in order to optimise surgical safety and efficacy.”	7 (6-8)	6	9	23	0	No

2. To what extent do you agree/disagree that the following are components of digital surgery

	Median (IQR)	1-3	4-6	7-9	Prefer not to answer	Consensus
Cloud computing	7 (5.25-8)	1	11	26	0	No
NEW: Computer assisted surgical training	8 (7.25-9)	0	4	34	0	Yes
NEW: Surgical decision support systems	9 (8-9)	0	3	35	0	Yes
NEW: Cognitive robotics	8 (6.25-9)	0	10	28	0	Yes
NEW: Data visualisation	8 (7.25-9)	0	5	33	0	Yes

3. To what extent do you agree/disagree that the following are benefits of digital surgery

	Median (IQR)	1-3	4-6	7-9	Prefer not to answer	Consensus
NEW: Quantifying outcome (beyond survival and other standard outcome measures; e.g. tissue injury)	8 (8-9)	0	4	34	0	Yes
NEW: Understanding and improving team dynamics	7.5 (6-9)	4	7	27	0	Yes
NEW: Understanding benefits and limitations of surgical strategies	8 (7-8.75)	4	5	29	0	Yes

4. Please rate how important you consider the following issues concerning data storage and security and AI in surgery

	Median (IQR)	1-3	4-6	7-9	Prefer not to answer	Consensus
Costs of data storage	7 (6.25-8)	4	6	28	0	Yes

5. Please rate how important you consider the following issues concerning data sharing and AI in surgery

	Median (IQR)	1-3	4-6	7-9	Prefer not to answer	Consensus
Adherence to present data rules can hamper competitiveness	7 (6-8)	0	11	27	0	Yes
Lack of motives for surgeons to share data	7 (7-8)	1	7	30	0	Yes

6. To what extent do you agree/disagree with the following statements about the consent process for surgical AI applications

	Median (IQR)	1-3	4-6	7-9	Prefer not to answer	Consensus
Consent should incorporate not only the patient but also the wider surgical team	7 (6-8)	3	11	24	0	No
NEW: Consent procedures should delineate if private (for profit) or public academic entities will access the data	8 (6-9)	6	5	26	1	No

7. Please rate how important you consider the following issues concerning transparency and public trust and AI in surgery

	Median (IQR)	1-3	4-6	7-9	Prefer not to answer	Consensus
Failure to produce an effective system to date	7 (6-8)	4	7	27	0	Yes
Lack of engagement with the public to date	7 (6.25-8)	0	10	28	0	Yes
Lack of existence of neutral body looking at public trust in AI	7 (6-8)	3	11	24	0	No
Fear of AI reinforcing biases in datasets	7 (6.25-8)	1	9	28	0	Yes

8. Please rate how important you consider the following issues concerning the law and AI in surgery

	Median (IQR)	1-3	4-6	7-9	Prefer not to answer	Consensus
Lack of standardisation of terminology concerning AI in law	8 (7-8)	1	6	30	1	Yes
Regulations concerning international data transfer unclear	8 (7.25-8.75)	0	4	34	0	Yes
Differing data laws between different countries	8 (7-8)	0	2	36	0	Yes

9. Please rate how important you consider the following issues concerning commercial partnerships and AI in surgery

	Median (IQR)	1-3	4-6	7-9	Prefer not to answer	Consensus
Handling inequality of power between involved parties	7 (6.25-8)	3	7	28	0	Yes
Patients may be less inclined to give data if commercial companies involved	7 (6-8)	3	11	24	0	No
Restriction on physician autonomy	7 (6-8)	4	11	23	0	No
Vendor lock-in	7 (6-8)	2	12	23	1	No
Defining force majeure (hospital/industry fails to meet contractual obligations due to unforeseen circumstances)	6.5 (5-7)	1	18	19	0	No

10. Please rate how important you consider the following barriers to developing AI in surgery

	Median (IQR)	1-3	4-6	7-9	Prefer not to answer	Consensus
Lack of international collaboration	7 (6-8)	2	10	26	0	No
Lack of funding into surgical AI projects	7 (6-9)	3	8	27	0	Yes

11. Please rate how important you consider the following barriers to deploying AI in surgery

	Median (IQR)	1-3	4-6	7-9	Prefer not to answer	Consensus
Medical workforce resistance	7 (6-8)	4	7	27	0	Yes
Resistance from hospital management	7 (6-8)	2	9	27	0	Yes
Hesitancy concerning cloud technologies	7 (6-8)	1	14	23	0	No
Insufficient cybersecurity measures	8 (7-8.75)	1	3	34	0	Yes
Network connectivity issues	7 (6-9)	1	11	26	0	No
Lack of public trust in AI	7 (6-8)	2	11	25	0	No

12. Please rate how important you consider the following barriers to monitoring AI in surgery

	**Median (IQR)**	**1-3**	**4-6**	**7-9**	**Prefer not to answer**	**Consensus**
Lack of resource and personnel dedicated to task	8 (7-9)	0	4	34	0	Yes
Costs associated with monitoring	7 (7-9)	0	5	33	0	Yes
No capability for remote monitoring at present	6.5 (6-7)	2	17	19	0	No
Divide between those monitoring and developing surgical AI systems	7 (7-8)	0	9	29	0	Yes

Supplement Note 4: List of consensus statements

1. Concerning digital surgery:

a. The definition of digital surgery is unclear
b. Digital surgery concerns pre-operative, peri-operative and post-operative aspects of surgery
c. Digital surgery is not restricted to the operating theatre but incorporates other aspects of surgery such as training, diagnosis and investigation

2. Components of digital surgery

a. Data capture
b. Data annotation
c. Data visualisation
d. Data storage
e. Data analysis
f. Artificial intelligence
g. Machine learning
h. Augmented or virtual reality
i. Computer vision
j. Advanced imaging systems
k. Robotics
l. Cognitive robotics
m. Surgical decision support systems
n. Surgical navigation systems
o. Computer assisted surgical training
p. Telesurgery
q. Remote monitoring
r. Sensor technology
s. Electronic health records
t. Digital patient pathways

3. Benefits of digital surgery

a. Improving diagnostics
b. Improving surgical efficiency
c. Improving clinical outcomes
d. Improving cost effectiveness
e. Improving patient care
f. Delivering patient specific treatment
g. Allowing pre-operative treatment planning
h. Identifying patient deterioration more promptly (an 'early warning system' for surgeons)
i. Error prediction
j. Error detection
k. Automation of surgical processes
l. Providing decision support to the surgeon
m. Reducing cognitive load on the surgeon
n. Standardisation of surgical processes
o. Evaluating surgeon performance
p. Improving surgeon ergonomics and health

q.	Accelerating surgical education
r.	Quantifying outcome (beyond survival and other standard outcome measures; e.g. tissue injury)
s.	Understanding benefits and limitations of surgical strategies
t.	Understanding and improving team dynamics

4. Issues concerning data access and AI in surgery

a.	Lack of infrastructure for data acquisition
b.	Data is not readily available in a digital format
c.	Lack of data quality standards
d.	Lack of data annotation standards
e.	Lack of data formatting standards
f.	Determining appropriate access to data
g.	Governance processes at present are overcomplicated and obstructive
h.	Lack of reliable datasets
i.	Lack of interoperability between different devices and systems

5. Issues concerning data storage and security and AI in surgery

a.	Appropriate encryption
b.	Lack of structure in data storage
c.	Hospitals currently lack the technical ability for data storage
d.	Costs of data storage
e.	Institutions are not equipped and under resourced to perform appropriate cybersecurity
f.	Implications of data breaches are poorly defined

6. Issues concerning data sharing and AI in surgery

a.	No guidelines concerning ownership of data
b.	Adherence to present data rules can hamper competitiveness
c.	No consensus on data sharing formats
d.	Data sharing across international boundaries is problematic
e.	Legal requirements concerning data sharing are unclear
f.	Lack of motives for surgeons to share data

7. Issues concerning privacy and confidentiality and AI in surgery

a.	Appropriate anonymisation of data
b.	Determining patient agreements for data sharing
c.	Ensuring public trust on data sharing
d.	Ensuring data is not used for other purposes if held
e.	Lack of education among surgical teams about the significance of data they are collecting
f.	The surgeon's right to privacy and influence on behaviour within the Operating Room

8. Issues concerning consent and AI in surgery

a.	Difficulties with consent if we do not know the future applications
b.	Incentivising patients to share data
c.	Management of a patient who chooses to withdraw consent
d.	Educating patients so consent is more informed

e. Ensuring patients fully understand what is being asked
f. Differing requirements of consent between countries
g. Rights of the surgeon and the wider surgical team to opt out

9. Concerning the consent process for surgical AI applications:

a. Consent procedures should delineate the extent of data collection
b. Consent procedures should delineate who will access the data
c. Consent procedures should explain why the data will be collected
d. Consent procedures should allow data collection for future or unknown applications
e. Patients should be consented separately should commercial partners have access to their data
f. There should be a standardised methodology for consenting patients to share their data

10. Issues concerning transparency and public trust and AI in surgery

a. Lack of explainability due to opaqueness of surgical AI systems
b. Failure to produce an effective system to date
c. Mandatory reporting of outcomes regardless of success/failure
d. Lack of engagement with the public to date
e. Lack of education around AI among the public
f. Fear of AI reinforcing biases in datasets

11. Issues concerning the law and AI in surgery

a. Lack of standardisation of terminology concerning AI in law
b. Law adapting behind technology curve and not fit for purpose
c. Lack of dedicated regulations concerning digital clinical data
d. Legal bases for data collection are unclear
e. Unclear who holds responsibility for data integrity under law
f. Data ownership model is unclear under the law
g. Lack of clarity legally concerning permissions to share data
h. Legal rights to privacy for the surgeon unknown
i. Regulations concerning international data transfer unclear
j. Differing data laws between different countries
k. Lack of education among stakeholders concerning data law
l. Poor availability of data law expertise within healthcare facilities
m. Ownership of intellectual property under law

12. Issues concerning litigation and liability and AI in surgery

a. Determining lines of responsibility – who is responsible if AI fails?
b. Determining liability if surgeons do not follow AI decision support tools
c. Effects of surgical AI on medical indemnity/insurance
d. Consequences of increased recording of data and its potential future use to determine medical negligence
e. Lack of regulations, at present, concerning litigation and liability

13. Issues concerning commercial partnerships and AI in surgery

a. Business model between hospitals and commercial companies not defined

b.	Lack of framework or experience within the majority of institutions for the setting up of fair partnerships
c.	Data sharing protocols not defined
d.	Differing motives between hospitals and commercial companies
e.	Handling inequality of power between involved parties
f.	Restriction on hospitals to report results

14. Barriers to developing AI in surgery

a.	Lack of digitisation in hospitals
b.	Hospital IT systems not fit for purpose
c.	Insufficient data availability
d.	Lack of shared ontology for annotation
e.	Burden of annotation
f.	Lack of data registry and platform standards
g.	Lack of standards in data formatting methods
h.	Lack of data quality standards
i.	Insufficient expertise in surgical AI
j.	Identification of the right problems to solve
k.	Poor interoperability between AI systems and embedded technology in the OR
l.	Difficulties in sharing data between multiple centres
m.	Lack of funding into surgical AI projects
n.	No success stories so far to drive field
o.	Priorities of clinicians and AI scientists not aligned
p.	Lack of knowledge of how to perform trials which demonstrate safety and utility of AI systems

15. Barriers to deploying AI in surgery

a.	Costs of setting up infrastructure
b.	Medical workforce resistance
c.	Resistance from hospital management
d.	Hindering of process due to bureaucratic processes
e.	Challenges in getting contractual relationships established
f.	Reimbursement or business model not clearly defined
g.	Lack of prioritisation given to new technology
h.	Institutional aversion to sharing patient data
i.	Inability to demonstrate safety or clinical benefit to stakeholders
j.	Insufficient cybersecurity measures
k.	Difficulties of integrating AI systems with existing IT infrastructure
l.	Variation in hospital IT systems
m.	Regulatory requirements are unclear at present
n.	Lack of framework for consenting and obtaining data
o.	Lack of AI literacy among surgical teams

16. Barriers to monitoring AI in surgery

a.	Unclear who should be responsible for monitoring
b.	Lack of resource and personnel dedicated to task
c.	Costs associated with monitoring
d.	Lack of standardised outcome measures for monitoring

e.	Difficulties in quantifying improvement
f.	Lack of prioritisation given to monitoring at present
g.	Divide between those monitoring and developing surgical AI systems

17. Technical future research goals for surgery in AI

a.	Standardisation of surgical data science platforms for data sharing and annotation
b.	Shared ontology for data annotation
c.	Dealing with unlabelled or weakly labelled data
d.	Uptake of common communication standard for surgical data
e.	Effective data collection systems
f.	Generation of open-source datasets
g.	Interoperability between different devices and systems
h.	Improving explainability of AI algorithms
i.	Identifying inequalities in underlying datasets

18. Clinical future research goals for surgery in AI

a.	Define most suitable use cases/applications for surgical AI
b.	Develop core outcomes, reporting and measurement sets relevant to AI in surgery
c.	Develop framework for introduction and evaluation of AI in surgery
d.	Determine trial methodology for assessment of surgical AI
e.	Encourage surgeons to share data
f.	Standardisation of processes

19. Organisational future research goals for surgery in AI

a.	Demonstrate impact of surgical AI systems
b.	Establish a model business plan with industry
c.	Improve public trust and education in AI
d.	Encourage interdisciplinary education – clinicians should understand basic AI, AI scientists should understand clinical needs
e.	Legal framework for introduction and monitoring of AI surgical systems
f.	Define impact of surgical AI systems on litigation and liability
g.	Organisation of task force involving all relevant stakeholders to define best practice standards for surgical AI
